# Optimization of Brilliant Blue R photocatalytic degradation by silver nanoparticles synthesized using *Chlorella vulgaris*

**DOI:** 10.1007/s11356-024-34967-3

**Published:** 2024-09-18

**Authors:** Agnieszka Sidorowicz, Giacomo Fais, Francesco Desogus, Francesco Loy, Roberta Licheri, Nicola Lai, Antonio Mario Locci, Alberto Cincotti, Roberto Orrù, Giacomo Cao, Alessandro Concas

**Affiliations:** 1 Interdepartmental Centre of Environmental Science and Engineering (CINSA), University of Cagliari, Via San Giorgio 12, 09124 Cagliari, Italy; 2 Department of Mechanical, Chemical and Materials Engineering, University of Cagliari, Via Marengo 2, 09123 Cagliari, Italy; 3 Department of Biomedical Sciences, University of Cagliari, Cittadella Universitaria, SS 554, Km 4.5, 09042 Monserrato, Italy; 4 Center for Advanced Studies, Research and Development in Sardinia (CRS4), Loc. Piscina Manna, Building 1, 09050 Pula, CA Italy

**Keywords:** Silver nanoparticles, *Chlorella vulgaris*, Photocatalytic remediation, Brilliant Blue R, Dye degradation

## Abstract

Synthesis of silver nanoparticles (Ag NPs) using microalgae is gaining recognition for its environmentally friendly and cost-effective nature while maintaining high activity of NPs. In the present study, Ag NPs were synthesized using a methanolic extract of *Chlorella vulgaris* and subjected to calcination. The X-ray diffraction (XRD) analysis showed a crystalline nature of the products with Ag_2_O and Ag phases with an average crystalline size of 16.07 nm before calcination and an Ag phase with 24.61 nm crystalline size after calcination. Fourier transform infrared spectroscopy (FTIR) revealed the capping functional groups on Ag NPs, while scanning electron microscopy (SEM) displayed their irregular morphology and agglomeration after calcination. The organic coating was examined by energy-dispersive X-ray spectroscopy (EDX) and thermogravimetric (TGA) analyses, confirming the involvement of the metabolites. The UV–Vis analysis showed a difference in optical properties due to calcination. Synthesized Ag NPs were applied for the photodegradation of hazardous dye Brilliant Blue R in visible light. Different values of light intensity, catalyst dose, initial dye concentration, and pH were tested to identify the optimal set of operating conditions. The highest degradation efficiency of 90.6% with an apparent rate constant of 0.04402 min^−1^ was achieved after 90 min of irradiation in the highest tested catalyst dosage.

**Supplementary Information:**

The online version contains supplementary material available at 10.1007/s11356-024-34967-3.

## Introduction

The discharge of dyes from various industries into water bodies is a major source of environmental pollution, which can affect aquatic biota as well as humans. It is estimated that more than 700,000 tons of synthetic dyes are produced annually, with about 15% released to the environment after processing (Fito et al. 2023). Once released into a water body, dyes can block solar light penetration, which reduces photosynthetic activity and inhibits the growth of aquatic biota (Faizal et al. 2023). In addition, due to their recalcitrant nature, they are resistant to degradation, resulting in prolonged exposure and accumulation in living organisms (Emmanuel et al. 2023). As a result, they can cause allergies, skin irritation, respiratory disorders, mutagenicity, and carcinogenicity (He et al. 2022). Therefore, it is crucial to apply the proper wastewater treatment method before their discharge.

One of the dyes commonly used in the laboratory is Brilliant Blue R (BBR), known for its intense blue color, often utilized for protein gel staining to visualize and analyze protein bands. BBR is also largely used in textile industries. It belongs to the class of triphenylmethane dyes, which contain three phenyl rings linked to a central carbon atom (Zhao et al. 2021). In its structure, conjugated double bonds in the aromatic rings enable electron delocalization with resonance stabilization, which allows electronic charge distribution and prevents the formation of reactive sites (Duxbury 1993). Combined with a lack of highly labile functional groups, the removal of BBR requires specialized techniques for its degradation (Khataee and Kasiri 2010).

In an attempt to discover economical and environmentally friendly treatments, several biological methods have been researched (Dutta and Bhattacharjee 2022). The mechanism behind them involves the use of enzymes found in the cell structures of microorganisms (Shabir et al. 2022). However, the treatment requires a longer time in a controlled, optimally favorable environment. Another common method is adsorption, which involves the adhesion of dye molecules into adsorbent through physical or chemical interactions (Shabir et al. 2022). While short reaction time is the advantage of using materials such as activated carbon or zeolites, the pollutants are collected and transferred, but not eliminated from the environment (Shabir et al. 2022). A different method, combining both high efficiency and short time, is the use of advanced oxidation processes (AOPs) based on the production of hydroxyl free radicals with high oxidizing ability.

Photocatalysis has emerged as a promising method utilizing light energy to generate reactive oxygen species (ROS) that can degrade the dye into less toxic substances (Peramune et al. 2022). Among various photocatalysts, silver nanoparticles (Ag NPs) have attracted attention due to their strong light absorption in the visible region and prominent activity (Liu et al. 2023). Moreover, their synthesis process can be sustainable and environmentally friendly when the metabolites from organisms are used as reducing and stabilizing agents (Sidorowicz et al. 2023). Among the organisms investigated for the synthesis of nanoparticles, there are microalgae, owing to their rapid biomass increase and abundance of valuable metabolites (Sidorowicz et al. 2022). Microalgae offer several advantages in cultivation, making them an attractive option for various applications. Their rapid growth rates, often surpassing terrestrial plants, enable efficient biomass production, and they can thrive in diverse environments, including non-arable land and wastewater, reducing competition for valuable agricultural resources. Furthermore, microalgae can utilize various carbon sources, including carbon dioxide, contributing to carbon sequestration and potentially mitigating greenhouse gas emissions. Microalgae can be also grown in different wastewater with a consequent reduction of the need of synthetic fertilizers and the positive side effect of remediating the wastewaters (Concas et al. 2021a). The high lipid content in certain microalgae species makes them valuable for biofuel production (Concas et al. 2021b). Overall, the versatile cultivation characteristics of microalgae make them a promising and environmentally friendly resource for diverse applications. The study by Rajkumar et al. showed the potential of Ag NPs from *Chlorella vulgaris* to photocatalyze methylene blue dye degradation under sunlight irradiation (Rajkumar et al. 2021); however, to the best of our knowledge, the activity of Ag NPs from *C. vulgaris* extract against BBR dye has not been assessed so far.

From the perspective of the significance of biological synthesis, the *C. vulgaris* methanolic extract was utilized to synthesize Ag NPs. The material was also calcined to determine the influence of its organic content on the structure and activity. The product was tested for the first time for photocatalytic degradation of BBR dye in visible light, considering the influence of various parameters, such as light intensity, dye concentration, catalyst concentration, pH, and the presence of organic content on the photocatalytic action. Herein, the material characteristics-to-photocatalytic performance was evaluated, and the degradation mechanisms were explored for the optimization of the removal of hazardous BBR dye from the environment.

## Materials and methods

### Synthesis of nanoparticles

The *Chlorella vulgaris* (CCALA 902) culture was grown in Bold’s basal medium (BBM) supplemented with 60 mM NaHCO_3_ with 300 RPM stirring and irradiation of 58 W fluorescent lamps (Osram®) with 60 μmol/m^2^/s photon flux. The culture was kept for 30 days to acquire an adequate quantity of biomass. Then, it was centrifuged at 1500 RPM at 4 °C (Heraeus® Megafuge® 1.0R) to remove the medium, and the algal biomass was dried at room temperature.

In the next step, 0.9 g of dried residue was mixed with 54 mL of methanol (Merck® LiChrosolv® hypergrade). The flask was sonicated for 30 min (Soltec® Sonica® 2400 ETH S3) followed by stirring at 250 RPM (IKA® RH Digital Magnetic Stirrer) for the next 30 min to break down the cell walls and release the metabolites into the solvent. The leftover biomass was removed by filtration using 11 µm standard filtration paper (Whatman®), and then, the liquid was evaporated using a rotary evaporator (BUCHI Rotavapor™ R-210 Rotary Evaporator System) to discard about 70% of methanol. The concentrated extract was diluted with Milli-Q H_2_O (Millipore®, Milan, Italy) to the final volume of 180 mL, and it was used for the Ag NP synthesis process.

For Ag NP synthesis procedure, the prepared extract was heated to 85 °C and stirred at 250 RPM (IKA® RH Digital Magnetic Stirrer). When the temperature of the extract had reached 85 °C, 0.1 M of silver nitrate (Carlo Erba®) was added and, after 15 min, pH of the solution was increased up to 8 using 1.25 M NaOH. Starting from the moment of silver nitrate addition, the reaction continued for 1.5 h, and then, the solution was removed from the hot plate stirrer for maturation at room temperature. Then, the liquid was centrifuged at 4000 RPM at 8 °C (Heraeus® Megafuge® 1.0R), followed by the repeated washing of the residue in the two washing cycles with Milli-Q H_2_O (Millipore®, Milan, Italy). Next, Ag NPs were dried at 80 °C for 24 h, ground using mortar and pestle, and divided into two parts. One part was stored in an Eppendorf tube in the absence of light (before calcination — Ag NPs BC), and the other was calcined in a muffle furnace (Gelman Instrument®) for 2 h at 600 °C (after calcination — Ag NPs AC). After this, all samples were stored in the same conditions.

### Characterization

The structure of Ag NPs was studied by X-ray diffraction (XRD) using X-ray diffractometer (D8 Advance, Bruker AXS®). The scanning was performed with a diffraction angle between 12 and 90° at 0.1° per 10 s, at 40 kV and 30 mA using CuKα (*λ* = 1.54 Å) radiation. The existing phases in obtained Ag NPs were identified by using Diffrac.Eva software v.6.1.0.4 according to the COD database. The crystallite size was calculated from Debye–Scherrer’s formula after baseline correction.

Functional groups and important bonds existing in the Ag NPs were determined by Fourier transform infrared spectroscopy (FTIR) using FT/IR-6700 (Jasco, Tokyo, Japan) in the range of 500–4000 cm^−1^.

The surface morphology of prepared Ag NPs was studied by scanning electron microscopy (SEM) using Hitachi S4000 FEG HRSEM (Hitachi Ltd., Tokyo, Japan) operated at 20 kV. The image acquisition was performed with Quartz PCI software (Quartz Imaging Corporation, Vancouver, Canada). Prior SEM analysis, the samples were coated with 2 nm of platinum to enhance the contrast. The composition assessment of Ag NPs was examined through EDX analysis using UltraDry EDX Detector (Thermo Fisher Scientific®, Madison, WI, USA) and NSS3 software (Thermo Fisher Scientific®, Madison, WI, USA).

Thermal properties of Ag NPs were studied with thermogravimetric analysis (TGA) using differential thermal analyzer (TG/DSC) (NETZSCH® STA 409 PC) in air with airflow 100 mL/min and in a heating range 25–1000 °C at 10 °C/min.

The optical properties were examined by UV–Vis absorption measurements using CARY 50 spectrophotometer (Varian Inc., Australia) with a cell path length of 10 mm in the wavelength range of 200–750 nm. The direct bandgap energy was calculated from the Tauc relation (Eq. 1):

1 $${(\alpha h\nu )}^{2}=(h\nu -{E}_{g})$$

where *α* is the molar extinction coefficient, $h$ is the Plank’s constant, *ν* is the light frequency, and *E*_*g*_ is the band gap energy. The bandgap energy was calculated by linear fit extrapolation of the plot of (*αhν*)^2^ against energy.

### In situ photocatalysis

**Fig. 1:**
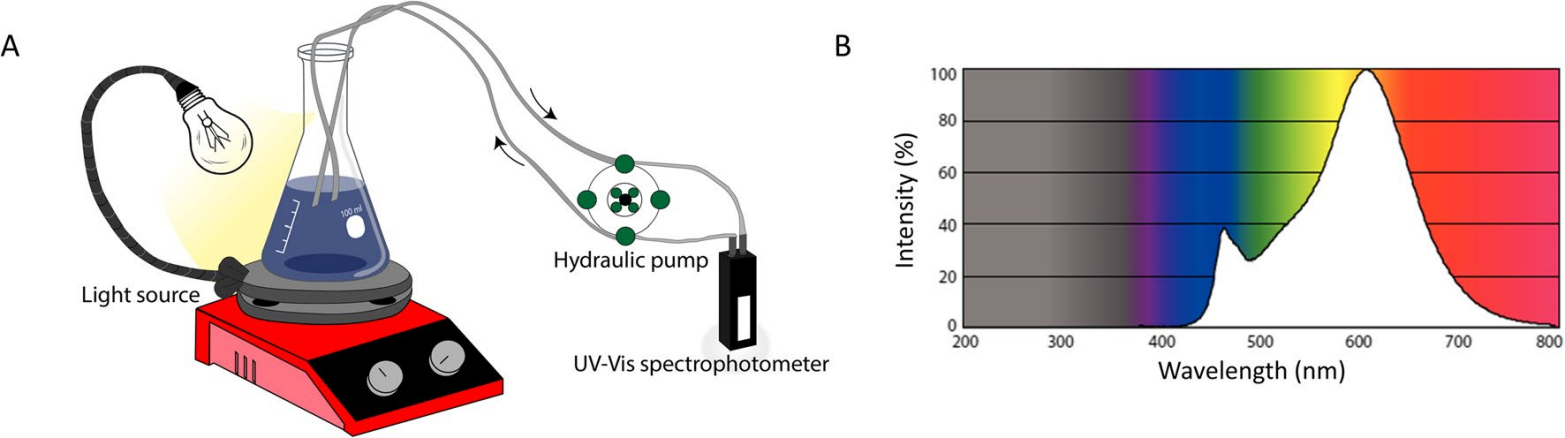
Scheme of the experimental setup for the photocatalytic degradation experiments. **A** In situ catalytic setup. **B** Emission spectrum of the light source provided by manufacturer

The scheme of the in situ photocatalytic setup is shown in Fig. 1A. First, calibration line was obtained by assessing the correlation between absorbance and concentration of the dye (Fig. S1). Before testing Ag NPs photocatalytic abilities, they were combined with 50 mL of ddH_2_O and sonicated for 15 min in a sonication bath (Soltec® Sonica® 2400 ETH S3) to ensure even dispersion. First, the baseline of the dispersed Ag NP solution was created. Next, the solution was mixed with different concentrations of BBR dye (Sigma-Aldrich®) in ethanol (500 mg/L). The flask was slowly stirred for 30 min without any exposure to light to achieve reaction equilibrium. Then, the light was switched on and the irradiation by a warm white 10.5 W LED bulb (Phillips) was measured with a luxmeter (HD2302.0 Delta-Ohm, Padua, Italy). The emission spectrum of the radiation source as supplied by the manufacturer is shown in Fig. 1B. The liquid was flowing continuously from the flask to a 10-mm flow-through cuvette attached to the UV–Vis Spectrophotometer (Cary 50, Varian®) and then back to the flask. The measurements were conducted in the range of 400–750 nm every 10 min. The experiments with the highest degradation efficiency were performed in triplicate to accurately evaluate the photocatalytic potential of the material.

The efficiency of the BBR dye degradation by Ag NPs was calculated as shown in Eq. (2):

2 $$\eta \left(\%\right)=\left(1-{~}^{C}\!\left/ \!{~}_{{C}_{0}}\right.\right)\cdot 100$$

where $\eta$ is the degradation efficiency, ${C}_{0}$ is the initial BBR dye concentration, and $C$ is the final concentration of the dye at the end of the degradation period.

According to the literature (Jiang et al. 2020), the data obtained were interpreted through the pseudo-first-order reaction model according to Eq. (3):

3 $$\frac{dC}{dt}=-{k}_{app}C$$

which, once solved, provides the following time evolution law for the dye concentration:

4 $$C={C}_{0}\text{exp}(-{k}_{app}t)$$

where $t (\text{min})$ is time and ${k}_{app} ({\text{min}}^{-1})$ is the apparent first-order rate constant. It should be noted that such model englobes the effects of both adsorption and photocatalysis in the same constant ${k}_{app}.$ Equation (4) was used to fit experimental data by tuning the value of ${k}_{app}$. The software OriginPro 2021© 9.8 was used for this purpose.

## Results and discussion

### Characterization

**Fig. 2:**
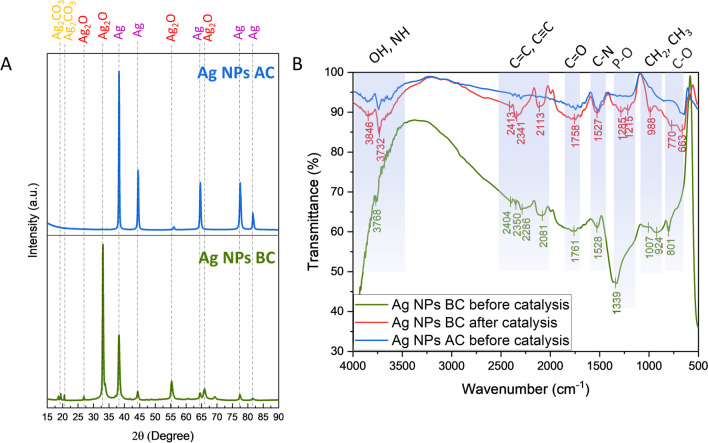
Crystallographic and spectroscopic analyses. **A** XRD. **B** FTIR. BC, before calcination; AC, after calcination

The XRD analysis was performed to determine the purity and crystallinity of the synthesized Ag NPs (Fig. 2A). The results of the analysis confirmed the presence of a highly pure and crystalline material, indicating a successful synthesis process. The identified phases in Ag NPs BC belong mostly to Ag_2_O (COD 1010486) and Ag (COD 1100136) with low intensity peaks at 2*θ* = 19–20° corresponding to Ag_2_CO_3_ (COD 4318190 and COD 4318187). After calcination, Ag NPs AC show the presence of only Ag phase (COD 9012961), which suggests the important role of organic content in stabilizing Ag NPs structure. Based on the XRD results, full width at half maximum (FWHM) values were calculated and used for Debye–Scherrer’s formula (Tab. S1–S2). The crystalline size for Ag NPs BC was determined as 16.44 nm for Ag_2_O and 15.70 nm for Ag with the average size of 16.07 nm. Calcination increased the crystalline size of Ag NPs AC to 24.61 nm.

The reported Ag NPs, synthesized using *C. vulgaris* and water as a solvent to extract metabolites, which resulted in the formation of only Ag phase in their structure (Soleimani and Habibi-Pirkoohi 2017; Mahajan et al. 2019; Rajkumar et al. 2021). The methanolic extract of *C. vulgaris* was studied previously for its high antioxidant activity owning to the presence of phenolic and flavonoids constituents as compared to the standard ascorbic acid (Pradhan et al. 2021). Moreover, the extraction technique is usually easier than applied to plants. Plant tissues typically have complex cell walls and organelles, necessitating methods such as homogenization and solvent extraction to release intracellular metabolites. In contrast, microalgae possess a simpler cell structure, often surrounded by a lipid-rich membrane, making them amenable to techniques like direct solvent extraction or mechanical disruption. The current results show the potential of *C. vulgaris* to obtain a variety of materials depending on composition of the solution to which the metal precursor is added. The organic content was further examined by FTIR analysis (Fig. 2B).

The FTIR technique was applied to study the behavior of the capping agents on Ag NPs before and after photocatalysis as well as before and after calcination. The results revealed an abundance of functional groups originating from the *C. vulgaris* extract such as OH, NH, C = C, C≡C, C = O, C-N, P-O, CH_2_, CH_3_, and C-O, with the highest intensity before calcination (Dilek (Yalcin) Duygu 2012; Mecozzi et al. 2012; Agazzi et al. 2020). After catalysis, the wavenumber values of the peaks shifted, which might be correlated with changes in the structure or organic molecules due to the interaction with radicals generated upon light irradiation. Moreover, the intensity of the peaks increased which further proves the active participation of the capping compounds in the photocatalytic process. The calcination process significantly increased the intensity of the peaks to around 95% which proves the efficient removal of organic content from the Ag NP surface.

**Fig. 3:**
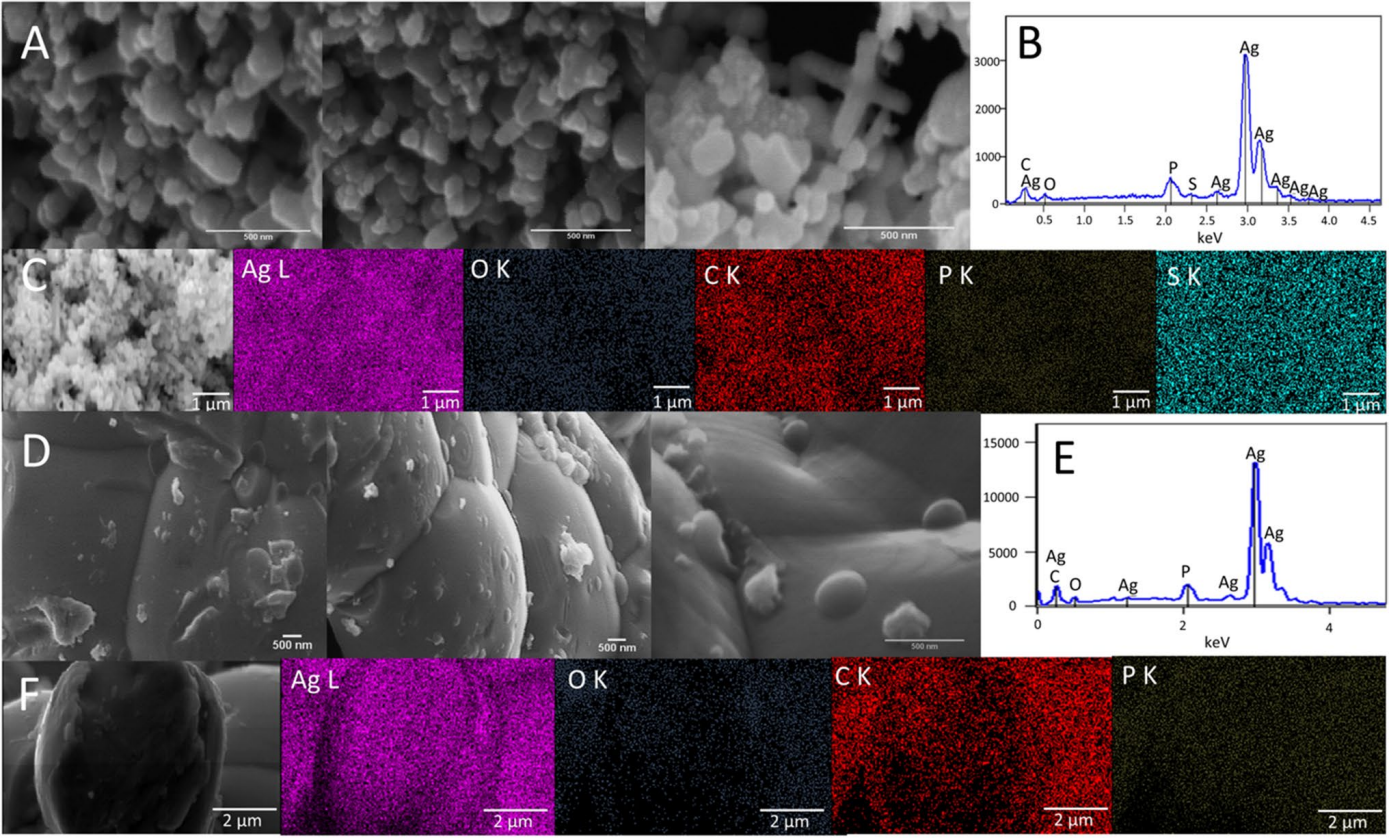
Microscopy analysis. **A** SEM of Ag NPs BC, **B** EDX spectrum of Ag NPs BC, **C** EDX mappings of Ag NPs BC, **D** SEM of Ag NPs AC, **E** EDX spectrum of Ag NPs AC, **F** EDX mappings of Ag NPs AC

The morphology of the obtained Ag NPs was observed using SEM–EDX analyses (Fig. 3). The Ag NPs BC exhibit irregular shapes with oval and ellipsoidal shapes. The elemental analysis revealed the presence of silver, oxygen, carbon, phosphorous, and sulfur in the structure with homogenic distribution. The Ag NPs AC showed the tendency to agglomeration or coalescence with more oval shape. The calcination resulted in the removal of sulfur from the Ag NPs while phosphorus remained as a part of their structure. Similarly, all detected elements showed homogeneous distribution. Partial removal of organic content from Ag NPs changed the morphology of the material which further proves the important role of organic content in stabilizing the structure and preventing agglomeration.

**Fig. 4:**
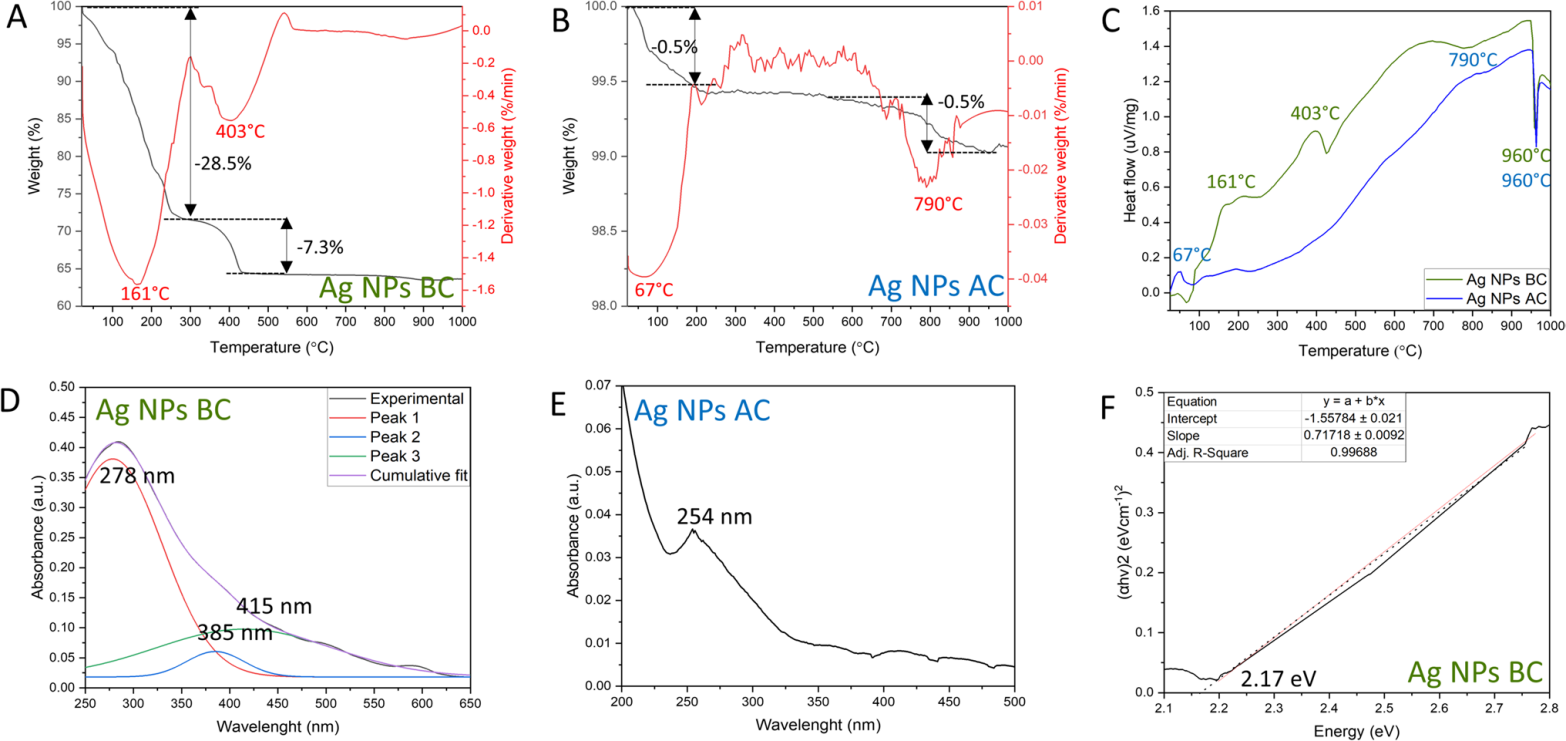
Thermal and optical properties of Ag NPs. **A** TG and DTG of Ag NPs BC, **B** TG and DTG of Ag NPs AC, **C** DTA of Ag NPs BC and Ag NPs AC, **D** UV–Vis spectrum of Ag NPs BC, **E** UV–Vis spectrum of Ag NPs AC, **F** Tauc plot of Ag NPs BC

Thermal properties of the Ag NPs were assessed by TGA analysis (Fig. 4A–C). For Ag NPs BC, two major weight losses can be observed: in the ranges 25–300 °C and 300–600 °C. The first weight loss with DTG peak at 161 °C, resulting in the loss of 28.5% weight, can be attributed to the water loss and decomposition of the temperature-sensitive organic compounds. The second weight loss with DTG peak at 403 °C led to decrease of 7.3% of the weight, which is probably due to the decomposition of the phenolic compounds present on Ag NPs BC surface (David and Moldovan 2020). Further increase in temperature did not change the weight significantly, with final weight at around 65%. In addition, DTA analysis proved the exothermic nature of the decomposition of the organic compounds described previously, with an endothermic peak at 960 °C, indicating the melting point of silver (Kis et al. 2022).

The Ag NPs AC subjected to the same analysis showed much smaller weight loss. In the 25—200 °C range, the weight decreased by 0.5% with DTG peak at 67 °C, which can be attributed to the physically adsorbed water molecules or oxidation of the remaining organic content on the Ag NPs AC surface (David and Moldovan 2020). The next weight loss occurred at around 700—900 °C, with the decrease of 0.5% and DTG peak at 790 °C, which is probably due to the decarbonization (Zin et al. 2023). At the end of the measurements, the final weight was recorded at around 99%. Similarly, to Ag NPs BC, the reactions were exothermic with endothermic melting silver point at 960 °C. Overall, the results show the high organic content of Ag NPs BC in comparison with Ag NPs AC, which supports the FTIR and EDX findings.

The optical properties of Ag NPs were studied by applying UV–Vis spectroscopy (Fig. 4D, E). Upon light excitation at specific wavelength, electrons in the conduction band undergo a collective oscillation known as a surface plasmon resonance. The effect results in strong scattering and absorption of light and can be an indicator of the potential photocatalytic activity. The UV–Vis spectrum of Ag NPs BC shows a broad absorbance band which, after deconvolution, reveals the presence of three separate peaks. The peaks at 278 nm and 385 nm probably belong to the metabolites present on the surface. The presence of additional peaks in the UV–Vis spectrum, due to the activity of organic molecules, has been observed before (Shankar et al. 2017). The characteristic surface plasmon resonance of Ag NPs was detected at 415 nm, and it is consistent with the literature, with values recorded in the 400–500-nm range (Ma et al. 2021). Furthermore, the Ag NPs BC can be photoactivated in the visible light spectrum.

After calcination, the absorbance values significantly decreased, which might be the effect of the structural defects and changes in the electronic structure induced by high temperature. The peak at 254 nm of Ag NPs AC is attributed to the electronic transition to metallic Ag (Baia and Simon 2007) which is consistent with the phase transition observed in XRD analysis. Thus, high-temperature treatment impaired the photocatalytic activity of Ag NPs.

Based on the UV–Vis spectrum, the band gap energy of Ag NPs BC was calculated (Fig. 4F). The parameter is defined as an energy difference between the highest occupied energy state of electrons in the valence band and the lowest unoccupied state of the conduction band. It determines the ability of the material to absorb light, generate electron–hole pairs, and initiate a photocatalytic reaction. The band gap energy of Ag NPs BC was calculated at 2.17 eV, and similar band gap values were reported previously (Mistry et al. 2022).

### Photocatalytic activity

**Fig. 5:**
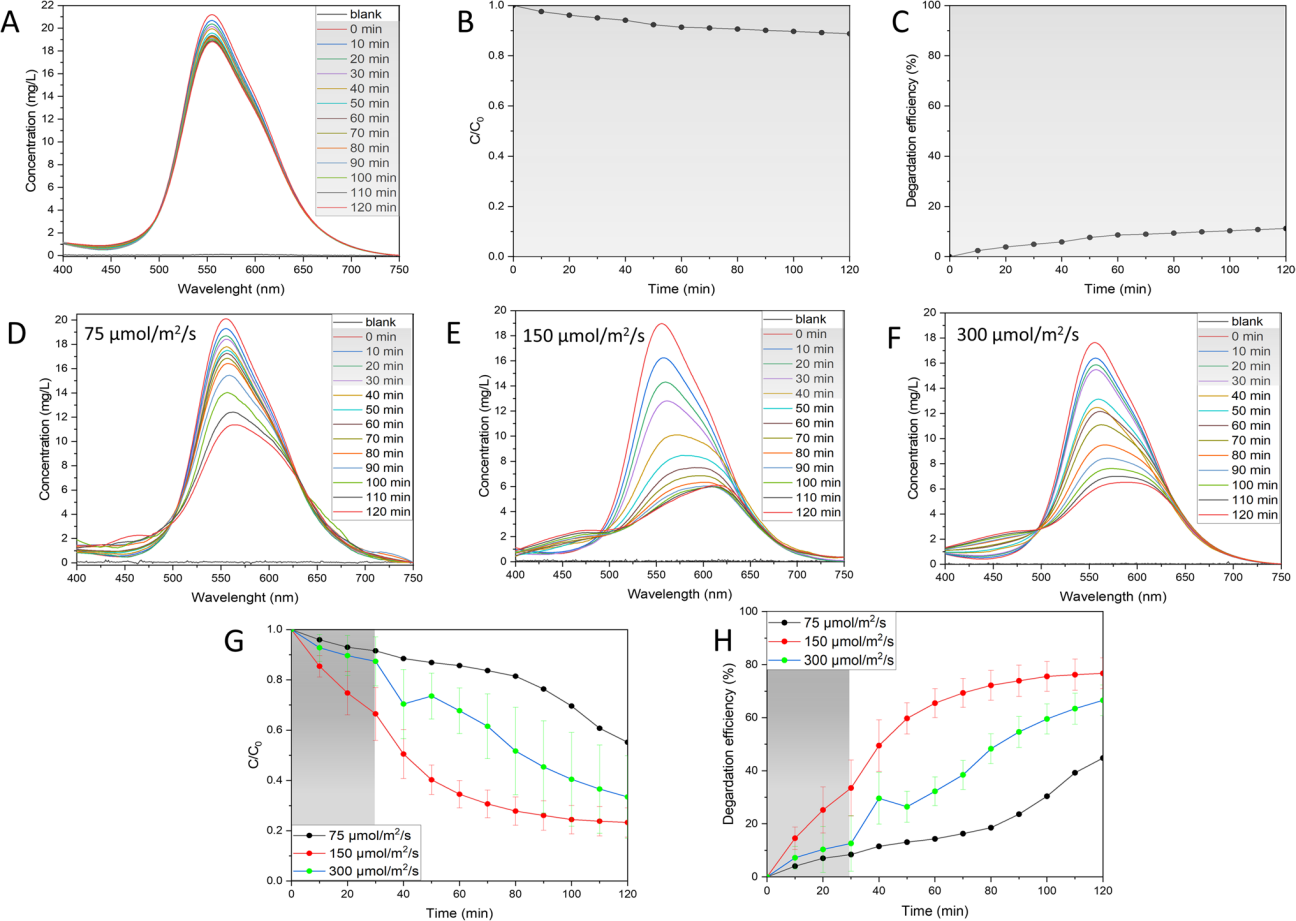
Influence of light on the photocatalytic activity. **A** UV–Vis spectral changes for the degradation of dye in the dark, **B** changes in dye concentration in the dark, **C** percentage degradation efficiency in the dark, **D–F** UV–Vis spectral changes in the varying light irradiation, **G** changes in dye concentration changes in the varying light irradiation, **H** percentage degradation efficiency changes in the varying light irradiation. Reaction conditions: dye concentration: 18–20 mg/L, Ag NPs BC concentration: 125 mg/L, pH 7

The correlation between absorbance and dye concentration is shown in Fig. S1. The dye degradation activity of Ag NPs BC was first assessed in the dark conditions (Fig. 5A–C; Fig. S2). The results showed a slight decrease in the dye concentration; however, after an initial period of 30 min, the concentration stabilized and started to reach equilibrium. The interactions between dye and Ag NPs BC are of physisorption nature, mainly due to weak van der Waals forces (Munagapati et al. 2023). At the end of the experiment, around 10% of the dye was degraded and *k*_*app*_ value was calculated as *k*_*app*_ = 0.00118 min^−1^ (Fig. S2). Based on the results obtained, the next experiments were performed after 30 min in dark conditions to allow the reaction to reach equilibrium and more precisely assess the effect of various factors on the photocatalytic performance of Ag NPs.

#### Influence of light

The first factor tested for photocatalytic activity was light intensity. In photocatalyst, the generation of electron and hole pairs is dependent upon the intensity or strength of the light that is directed onto it. When the intensity of light increases, there is a corresponding increase in the transfer of electrons from the valence band to the conduction band (Roy et al. 2023). This leads to an amplified generation of hydroxyl or oxygen radicals, playing a crucial role in the degradation of organic molecules (Roy et al. 2023). The concentration of BBR dye was measured in three different light intensities: 75, 150, and 300 µmol/m^2^/s (Fig. 5D–H; Fig. S3).

The lowest tested light intensity (75 µmol/m^2^/s) resulted in the lowest decrease in the concentration of the dye, with final degradation efficiency of around 40% and *k*_*app*_ value of 0.00402 min^−1^. The most significant increase of the degradation efficiency was recorded with increasing twice the light intensity, which resulted in the remarkable degradation efficiency of around 76.7%, with *k*_*app*_ equal to 0.01642 min^−1^. Further increase in light intensity showed a rapid change during the first 10 min of illumination, which could relate to the production of intermediates of BBR degradation products. At the end of the experiment, the degradation efficiency was recorded at around 66.5%, with *k*_*app*_ value of 0.01041 min^−1^. The decrease of the degradation efficiency in the highest light intensity might be due to the influence of heat provided to the system (Shabir et al. 2022). The results proved the important role of light intensity for BBR dye degradation using Ag NPs BC with higher number of absorbed light photons leading to the high likelihood of the BBR dye degradation taking place.

#### Influence of dye concentration

**Fig. 6:**
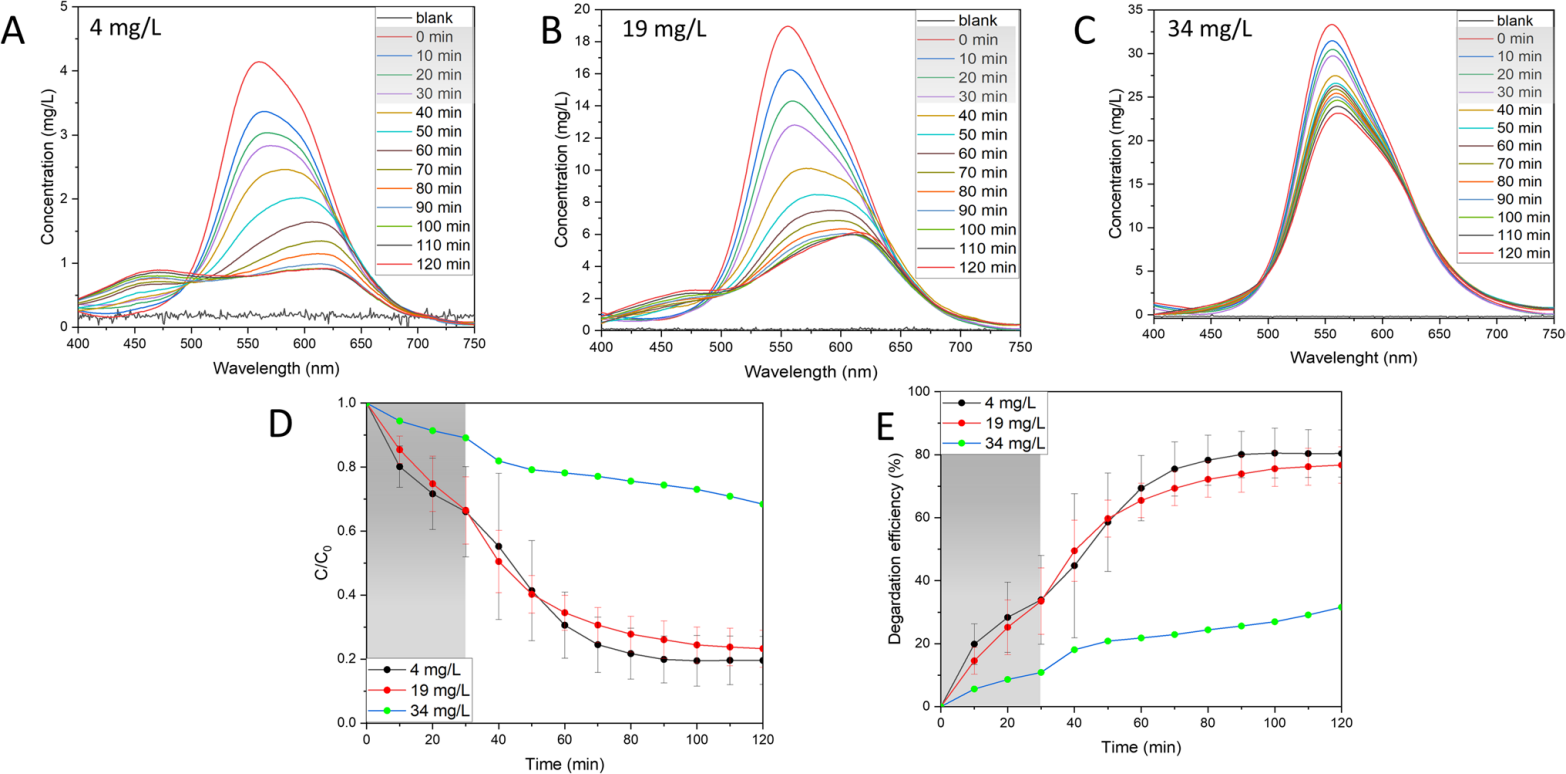
Influence of dye concentration on the photocatalytic activity. **A**–**C** UV–Vis spectral changes in the varying dye concentration, **D** changes in dye concentration, **E** percentage degradation efficiency. Reaction conditions: Ag NPs BC concentration: 125 mg/L, light intensity: 150 µmol/m^2^/s, pH 7

The light availability can also be influenced by changing the concentration of the BBR dye in the solution. The dye molecules can absorb light which results in fewer photons reaching the catalyst surface, leading to the decreased production of radicals. The effect of changing BBR dye concentration within range 4–34 mg/L is shown in the Fig. 6 and Fig. S4. As expected, the highest decrease in the BBR concentration was observed for the lowest tested BBR dye concentration of 4 mg/L with degradation efficiency around 80% and *k*_*app*_ value of 0.01983 min^−1^. The increase of the BBR dye concentration to 19 mg/L decreased the degradation efficiency to around 76.7% and *k*_*app*_ value 0.01642 min^−1^. The highest tested BBR dye concentration showed a degradation efficiency around 31.6% with *k*_*app*_ value 0.00314 min^−1^. Other than the easier light availability, the low degradation of the dye allows easier access of the molecules to the active sites on the catalyst surface and prevents agglomeration (Roy et al. 2023). Considering that the BBR dye is present in the wastewater due to many washing cycles (Chiarello et al. 2020), it shows the potential of Ag NPs BC for its efficient removal.

#### Influence of catalyst dosage

**Fig. 7:**
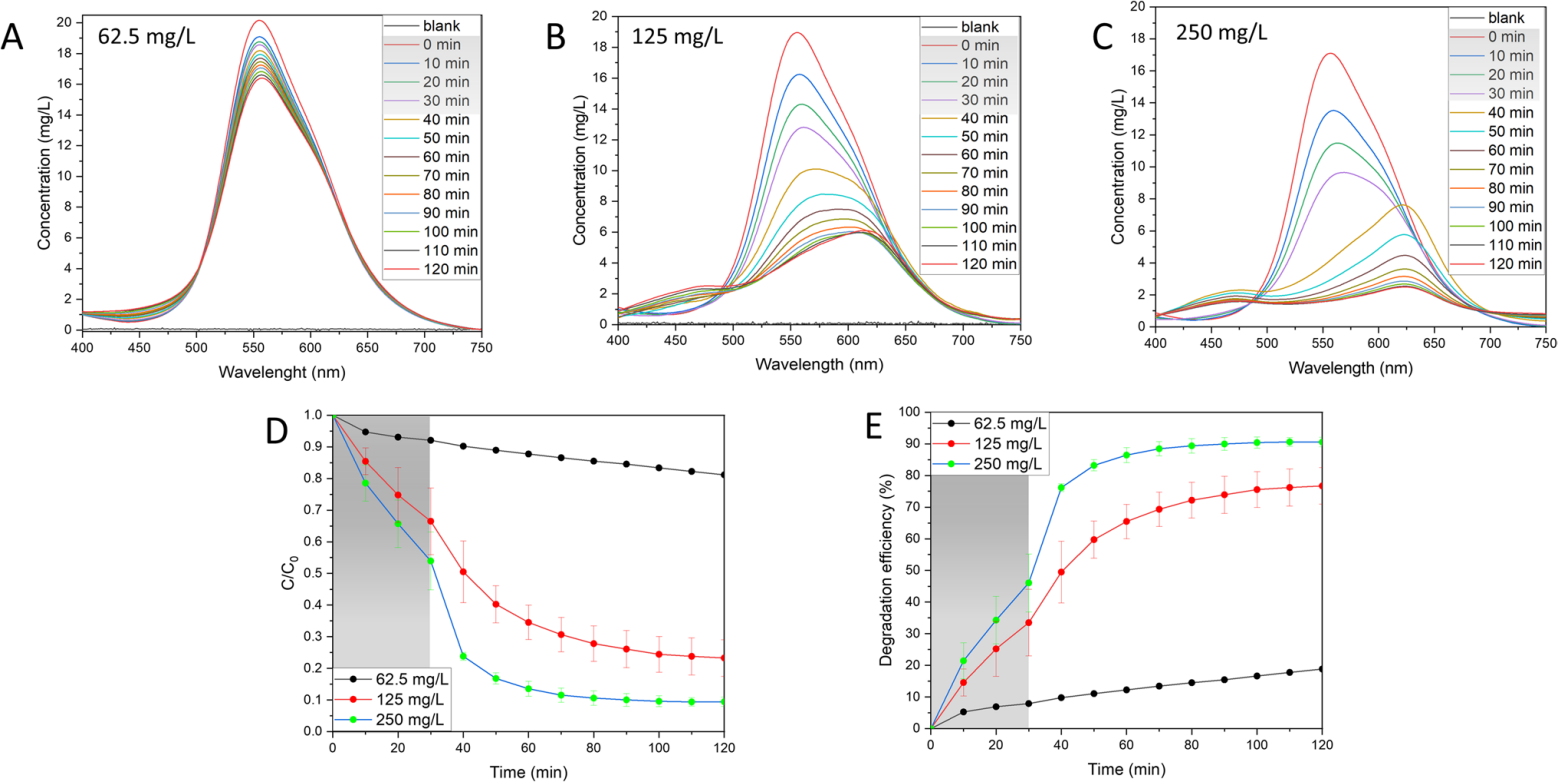
Influence of catalyst concentration on the photocatalytic activity. **A**–**C** UV–Vis spectral changes in the varying catalyst concentration, **D** changes in dye concentration, **E** percentage degradation efficiency. Reaction conditions: dye concentration: 17–20 mg/L, light intensity: 150 µmol/m^2^/s, pH 7

The influence of the availability of active sites on the catalyst surface has been tested by varying the catalyst dosage in the range 62.5–250 mg/L (Fig. 7; Fig. S5). The lowest tested concentration of Ag NPs BC resulted in only a slight decrease in BBR dye concentration with degradation efficiency of 18.8% and *k*_*app*_ value of 0.00144 min^−1^. Increasing twice the catalyst dosage significantly improved the photocatalytic performance with recorded degradation efficiency of around 76.7% and *k*_*app*_ value of 0.01642 min^−1^. The highest tested catalyst dosage of 250 mg/L revealed a rapid decrease in the BBR concentration after switching on the light with a peak shift which can be attributed to the formation of intermediates. The degradation efficiency of the reaction was observed at around 90.6% with *k*_*app*_ value of 0.04402 min^−1^. Based on the obtained results, the availability of the active sites on the Ag NPs BC surface plays a crucial role in the photocatalytic degradation of the BBR dye. However, it has been reported that the increase can be observed up to a certain limit as the excess of the catalyst might turn the solution more turbid, thus hindering the light penetration (Nawaz et al. 2023).

#### Influence of pH

**Fig. 8:**
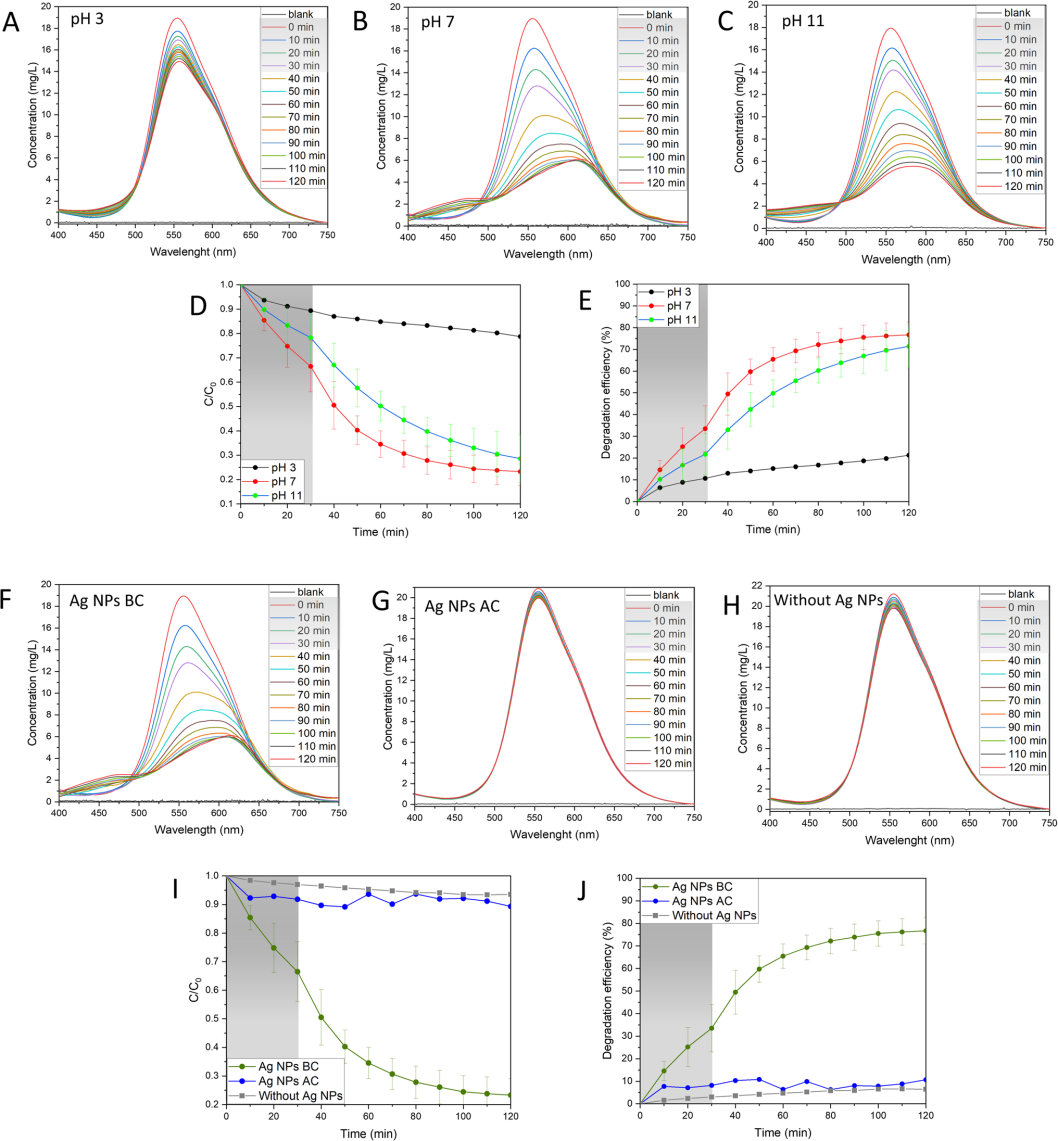
Influence of pH and calcination on the photocatalytic activity. **A**–**C** UV–Vis spectral changes in the varying pH, **D** changes in dye concentration in the varying pH, **E** percentage degradation efficiency in the varying pH. Reaction conditions: dye concentration: 18–19 mg/L, Ag NPs BC concentration: 125 mg/L, light intensity: 150 µmol/m^2^/s. **F** UV–Vis spectral changes before calcination, **G** UV–Vis spectral changes after calcination, **H** UV–Vis spectral changes without catalyst, **I** changes in dye concentration, **J** percentage degradation efficiency. Reaction conditions: dye concentration 19–21 mg/L, Ag NPs BC concentration 125 mg/L, light intensity 150 µmol/m^2^/s, pH 7

Another tested factor for the photocatalytic performance of Ag NPs BC was pH of the solution in the range 3–11 (Fig. 8A–E; Fig. S6). At pH 3, the photocatalytic degradation efficiency was recorded at 21.3% with *k*_*app*_ value of 0.00142 min^−1^, while pH 7 and 11 showed a degradation efficiency of around 76.7% and 71.4%, with *k*_*app*_ values of 0.01642 min^−1^ and 0.01311 min^−1^, respectively. The interpretation of pH effect is challenging due to its multiple roles, such as changing electrostatic interactions between the catalyst surface, solvent molecules, substrate, and formed radicals (Javanbakht and Mohammadian 2021). The alkaline medium might have facilitated the formation of hydroxyl radicals which improved the BBR dye removal process (Lin et al. 2022). The effect can continue up to a specific pH value when the negatively charged catalyst demonstrates Coulomb repulsion between the catalyst surface and present hydroxyl anions, diminishing the formation of radicals (Uma et al. 2019).

#### Influence of calcination

In the final set of experiments, the influence of the removal of the organic content by calcination was tested on photocatalytic activity (Fig. 8F–J; Fig. S7). The Ag NPs AC exhibited only a slight change in the BBR dye concentration with degradation efficiency around 10.7% and *k*_*app*_ value of 0.00245 min^−1^. In comparison, Ag NPs BC in the same conditions showed the degradation efficiency of 76.7% and *k*_*app*_ value of 0.01642 min^−1^. Without any catalyst, around 6.5% of the dye was degraded and *k*_*app*_ value was calculated at 0.00049 min^−1^. The effect of calcination was usually tested before on TiO_2_-Ag catalysts with the temperature affecting the morphology of TiO_2_ to improve its photocatalytic activity (Desiati et al. 2019; Nutescu Duduman et al. 2022). However, Khandan Nasab et al. reported the activity of Ag/Ag_2_O NPs in the UVA spectrum which might suggest that, after the calcination, the material requires higher energy wavelengths for photoactivation, increasing the cost of the degradation process (Khandan Nasab et al. 2020).

#### Mechanism of dye degradation

Based on the obtained results, the mechanism of BBR dye degradation is proposed as shown in the equations below (5–11) (Ahmed et al. 2020; Lin et al. 2022; Groeneveld et al. 2023). The mechanism of the dye degradation process is associated with the excitation of electrons (e^−^) from the valence band to the conduction band upon light irradiation (Eq. 5). After their transfer, positively charged holes (h^+^) react with water molecules producing H^+^ and $\cdot \text {OH}$ radical (6). The free electrons convert molecular oxygen into $\cdot {{\text{O}}_{2}}^{-}$ (7), which reacts with water molecules generating $\cdot \text {OOH}$ (8) rearranged into H_2_O_2_ (9). After the reaction of H_2_O_2_ with $\cdot {{\text{O}}_{2}}^{-}$, $\cdot \text {OH}$ is produced (10), which can also be generated as a result of interactions between hydroxyl ions and holes (11). The process generates $\cdot \text {OH}$, which plays an important role in the degradation of BBR dye.

5 $$\text{Photocatalyst surface }+ hv \to \text{ electron }({\text{e}}^{-}) +\text{ hole }({\text{h}}^{+})$$

6 $${\text{h}}^{+} + {\text{H}}_{2}\text{O }\to {\text{H}}^{+} + \cdot \text{OH}$$

7 $${\text{e}}^{-} + {\text{O}}_{2} \to \cdot {{\text{O}}_{2}}^{-}$$

8 $$\cdot {{\text{O}}_{2}}^{-} + {\text{H}}_{2}\text{O }\to \cdot \text{OOH }+ {\text{OH}}^{-}$$

9 $$2\!\cdot\! \text{OOH }\to {\text{H}}_{2}{\text{O}}_{2} + {\text{O}}_{2}$$

10 $${\text{H}}_{2}{\text{O}}_{2} + \cdot {{\text{O}}_{2}}^{-} \to \cdot \text{OH }+ {\text{O}}_{2} + {\text{OH}}^{-}$$

11 $${\text{OH}}^{-} +\text{ h}+ \to \cdot \text{OH}$$

The degradation of BBR dye was previously studied based on sonochemical process, which also involves generation of hydroxyl radicals (Rayaroth et al. 2015). There are two major pathways that were identified, using LC-Q-TOF–MS analysis, as a result of either OH^−^ addition or hydrogen abstraction and disproportionation/hydroxylation reaction. Then, the intermediates undergo many reactions, including oxidative cleavage, de-ethylation, or demethylation. There were 13 identified intermediates which can be subjected to further ring opening and other oxidative cleavages resulting in the mineralization reaction (Rayaroth et al. 2015). Moreover, the degradation pathway was also studied in the river water and yielded similar intermediate profile, which shows that the process is independent from presence of other ions or substances in river water (Rayaroth et al. 2017).

This study introduces a novel approach to dye degradation by leveraging Ag NPs that exhibit enhanced photocatalytic activity under visible light irradiation, making them more applicable to real-world environmental remediation. Although a previous study on utilizing methanolic extract of *C. vulgaris* revealed its potential to synthesize catalytically active NPs (Sidorowicz et al. 2024), the dye degradation performance of Ag NPs from *C. vulgaris* methanolic extract has not been described before. Unlike conventional NPs used in similar studies, the synthesis method using microalgal extract provides superior control over particle size, morphology, and surface properties, leading to unprecedented efficiency in the degradation of BBR dye at lower concentrations and shorter reaction times (Rajkumar et al. 2021). Furthermore, this research addresses a significant gap in the current literature by addressing the practical aspects of the operating conditions on the degradation mechanism and the fundamental understanding of nanoparticle-mediated dye degradation while also paving the way for developing more sustainable and efficient photocatalytic systems.

## Conclusions

The development of efficient and stable photocatalysts is of paramount importance in the pursuit of high-efficiency reaction systems for addressing polluted water environments. Indeed, the search for effective photoactive materials, capable of promoting photocatalytic reactions, has been the focus of extensive research efforts. In the present study, *C. vulgaris* methanolic extract was used to successfully synthesize Ag NPs, which were also subjected to calcination. The XRD findings showed the presence of Ag and Ag_2_O phase in NPs before calcination (Ag NPs BC), while NPs after calcination (Ag NPs AC) contained only Ag phase. The calculated crystalline size was 16.07 nm and 24.61 nm for Ag NPs BC and Ag NPs AC, respectively. The involvement of extract in the synthesis was confirmed by FTIR analysis, revealing the abundance of chemical groups on the Ag NPs BC surface. The SEM analysis showed the irregular morphology and the presence of elements from the extract was examined by EDX. The TGA findings showed the difference in the organic content of the prepared Ag NPs, and their optical properties were studied by UV–Vis analysis, revealing visible light activation of Ag NPs BC, with band gap energy of 2.17 eV.

The photocatalytic activity of Ag NPs BC against BBR dye was analyzed in the visible light wavelengths testing various factors such as light intensity, dye concentration, catalyst dosage, pH, and calcination of the catalyst. The highest degradation efficiency of 90.6% with *k*_*app*_ value of 0.04402 min^−1^ was archived by increasing the catalyst dosage. Finally, the BBR dye degradation mechanism was proposed. Thus, Ag NPs showed great potential as a photocatalyst against BBR dye, and they could also be explored for the degradation of other organic compounds.

## Supplementary Information

Below is the link to the electronic supplementary material.

Supplementary file1 (DOCX 950 KB)

## Data Availability

Data will be made available on request.
