# Supplementary material for: Optimization of Brilliant Blue R photocatalytic degradation by silver nanoparticles synthesized using Chlorella vulgaris
Source: Environ Sci Pollut Res Int. 2024 Sep 18;31(47):57765–77. doi: 10.1007/s11356-024-34967-3 (PMC11466998; doi:10.1007/s11356-024-34967-3)
Supplement: Supplementary file 1 — Supplementary file1 (DOCX 950 KB) [file 11356_2024_34967_MOESM1_ESM.docx]

Supplementary material

**Optimization of Brilliant Blue R photocatalytic degradation by silver nanoparticles synthesized using *Chlorella vulgaris.***

Agnieszka Sidorowicz^1^, Giacomo Fais^1,2^, Francesco Desogus^1,2^, Francesco Loy^3^, Roberta Licheri^1,2^, Nicola Lai^1,2^, Antonio Mario Locci^1,2^, Alberto Cincotti^1,2^, Roberto Orru’^1,2^, Giacomo Cao^1,2,4^ and Alessandro Concas^1,2,*^

^1^ Interdepartmental Centre of Environmental Science and Engineering (CINSA), University of Cagliari, Via San Giorgio 12, 09124 Cagliari, Italy.

^2^ Department of Mechanical, Chemical and Materials Engineering, University of Cagliari, Via Marengo 2, 09123 Cagliari, Italy.

^3^ Department of Biomedical Sciences, University of Cagliari, Cittadella Universitaria, SS 554, Km 4.5, 09042 Monserrato, Italy

^4^ Center for Advanced Studies, Research and Development in Sardinia (CRS4), Loc. Piscina Manna, Building 1, 09050 Pula (CA), Italy.

* correspondence should be addressed to: Prof. Alessandro Concas, Phone: +39 070 6755076, Fax: +39 070 6755057, e-mail: alessandro.concas@unica.it

Table S1 XRD values and crystalline size of the detected phases in Ag NPs BC.

| 2 Theta (Degree) | FWHM | Crystalline size (nm) | Detected phase |
| --- | --- | --- | --- |
| 33.05625 | 0.35137 | 23.58395281 | Ag_2_O |
| 38.27079 | 0.52924 | 15.88865158 | Ag |
| 44.32271 | 0.49076 | 17.47898325 | Ag |
| 55.35625 | 0.69112 | 12.98010399 | Ag_2_O |
| 64.54108 | 0.58953 | 15.93737457 | Ag |
| 65.96998 | 0.89933 | 10.53102607 | Ag_2_O |

Table S2 XRD values and crystalline size of the detected phases in Ag NPs AC.

| 2 Theta (Degree) | FWHM | Crystalline size (nm) | Detected phase |
| --- | --- | --- | --- |
| 38.28847 | 0.26019 | 32.32007473 | Ag |
| 44.4653 | 0.34024 | 25.22437749 | Ag |
| 64.61883 | 0.40804 | 23.03595174 | Ag |
| 77.56777 | 0.522 | 19.52362767 | Ag |
| 81.71249 | 0.45745 | 22.96078457 | Ag |


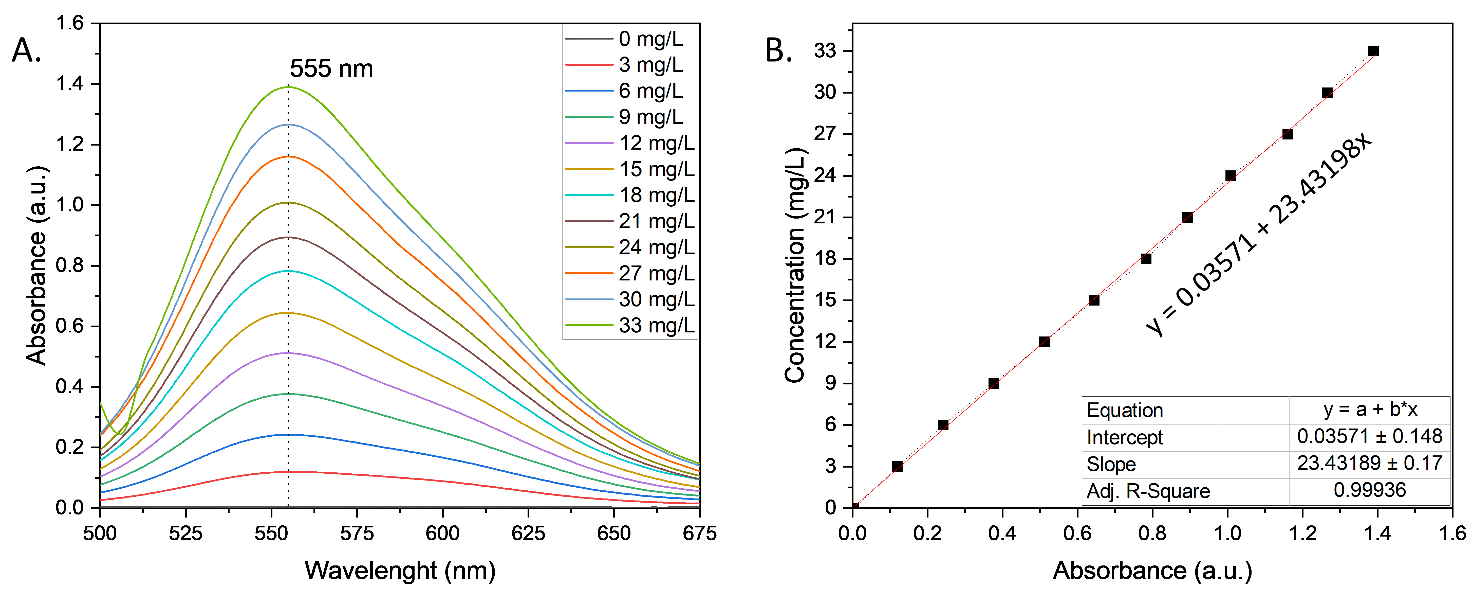


Figure S1 Calibration line (A) absorbance profile of BBR dye at different concentrations, (B) plot of concentration at the maximum absorbance peak at 555 nm.


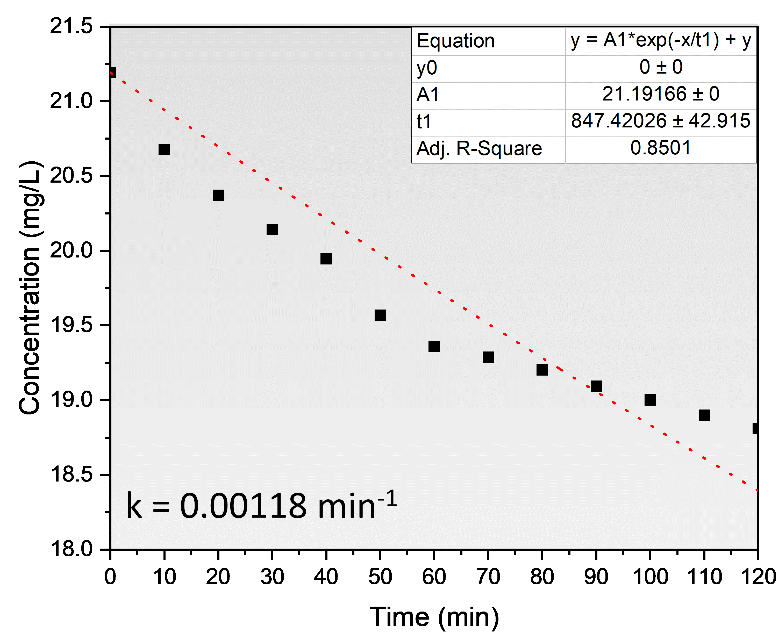


Figure S2 Rate constant in the dark conditions. Fitted parameters were calculated based on the dotted red plot.


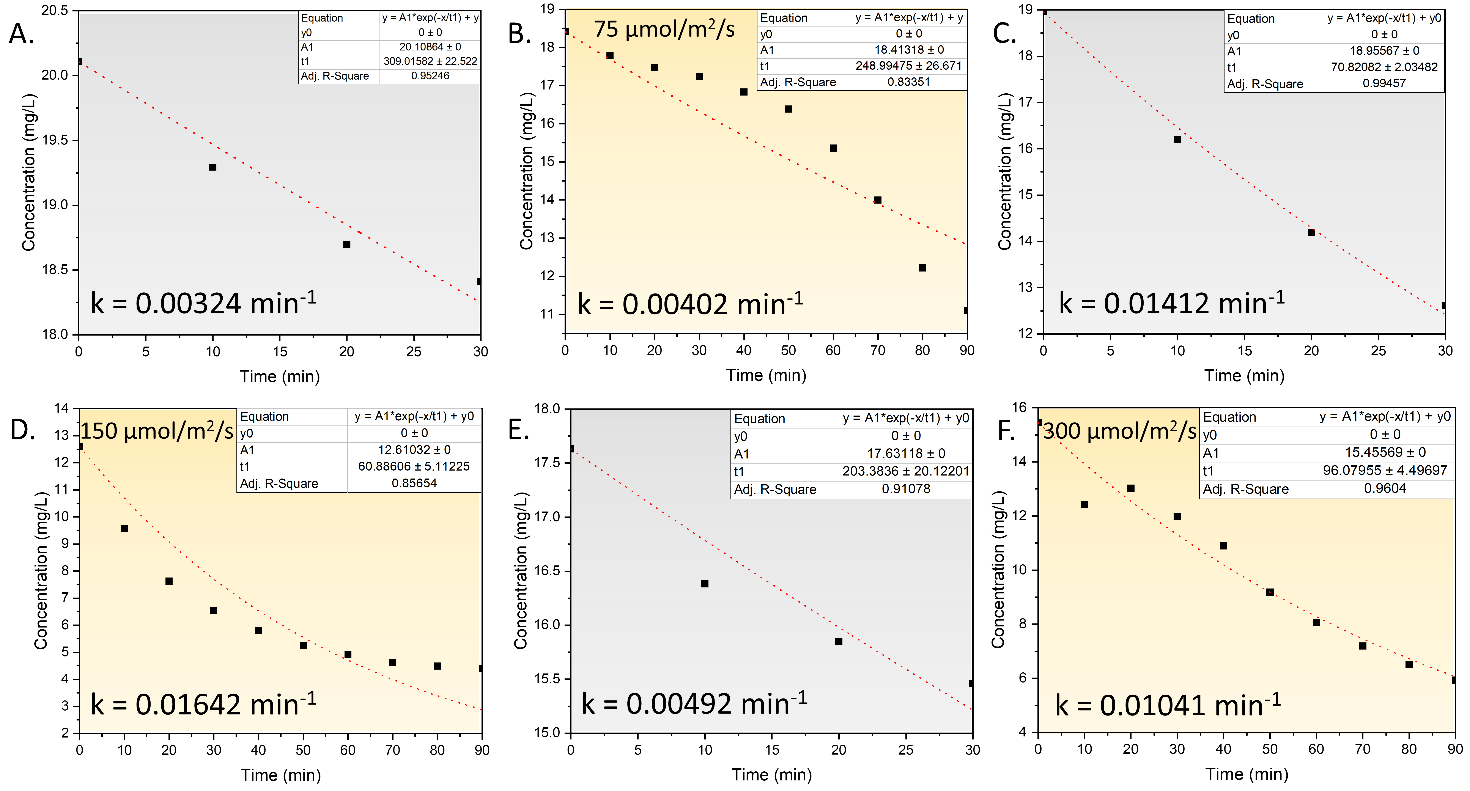


Figure S3 Rate constant at different light intensities, (A) dark, (B) 75 µmol/m^2^/s, (C) dark, (D) 150 µmol/m^2^/2, (E) dark, (F) 300 µmol/m^2^/s. Fitted parameters were calculated based on the dotted red plot.


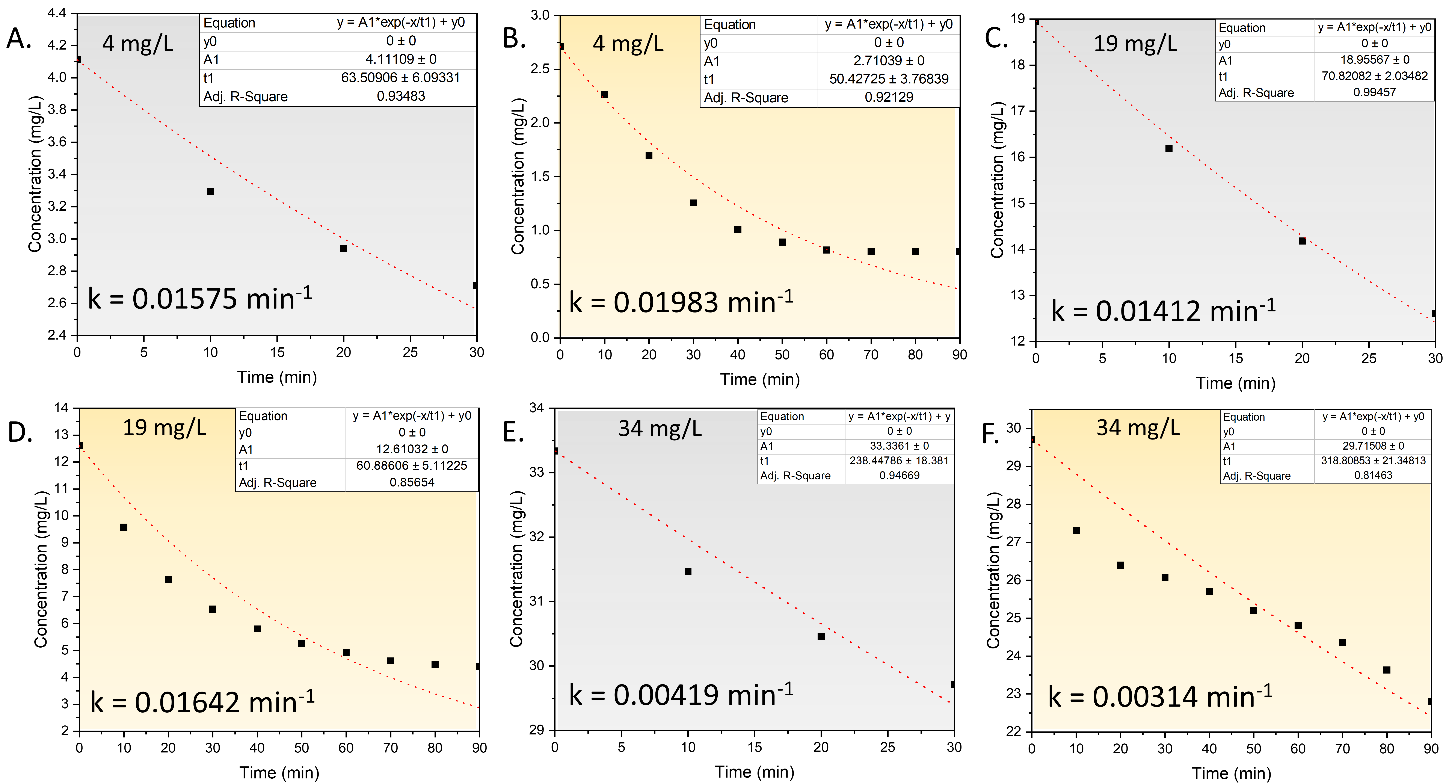


Figure S4 Rate constant at different dye concentrations (A) 4 mg/L in the dark, (B) 4 mg/L in the light, (C) 19 mg/L in the dark, (D) 19 mg/L in the light, (E) 34 mg/L in the dark, (F) 34 mg/L in the light. Fitted parameters were calculated based on the dotted red plot.


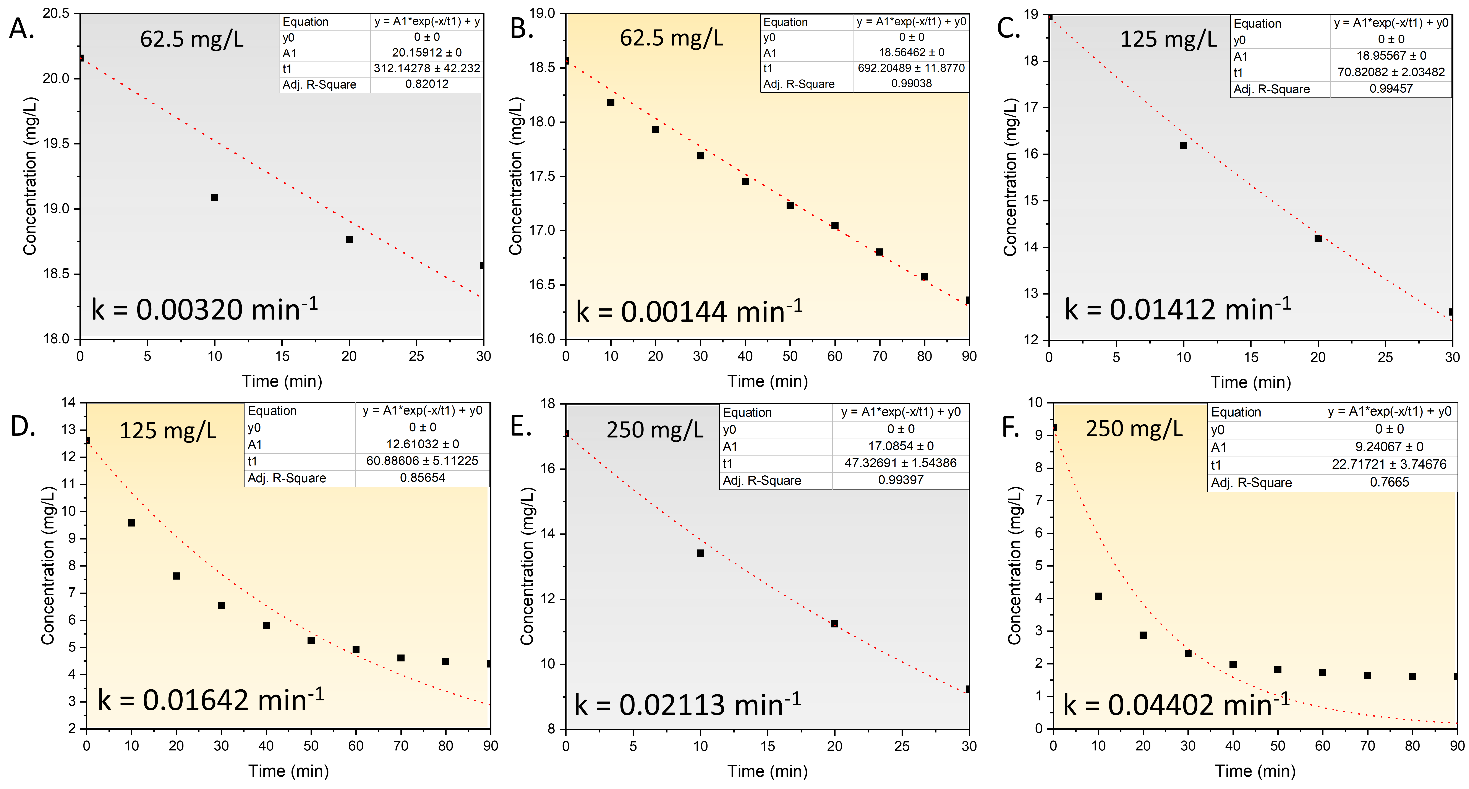


Figure S5 Rate constant in different catalyst loadings, (A) 62.5 mg/L in the dark, (B) 62.5 mg/L in the light, (C) 125 mg/L in the dark, (D) 125 mg/L in the light, (E) 250 mg/L in the dark, (F) 250 mg/L in the light. Fitted parameters were calculated based on the dotted red plot.


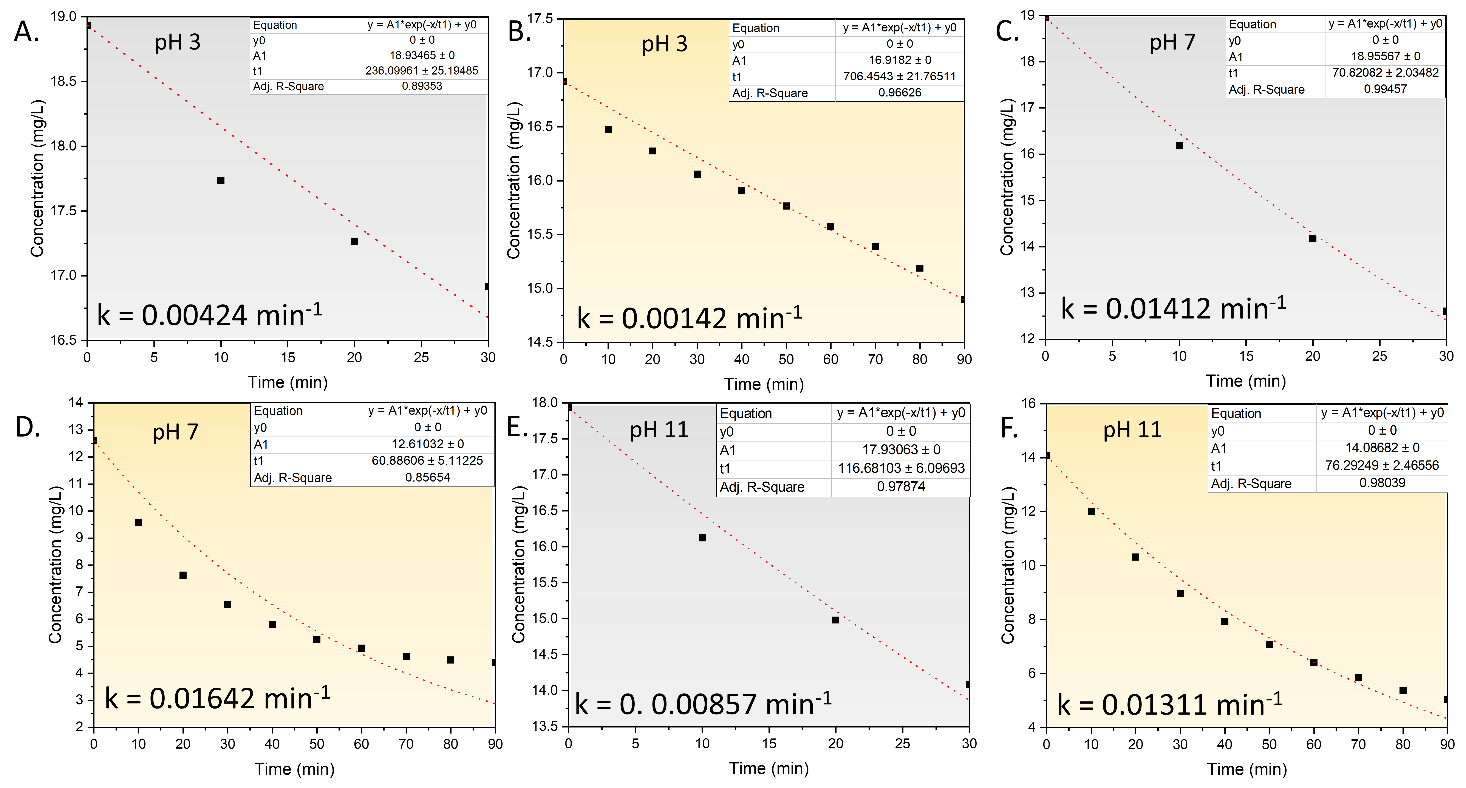


Figure S6 Rate constant in the different pH, (A) pH 3 in the dark, (B) pH 3 in the light, (C) pH 7 in the dark, (D), pH 7 in the light, (E) pH 11 in the dark, (F) pH 11 in the light. Fitted parameters were calculated based on the dotted red plot.


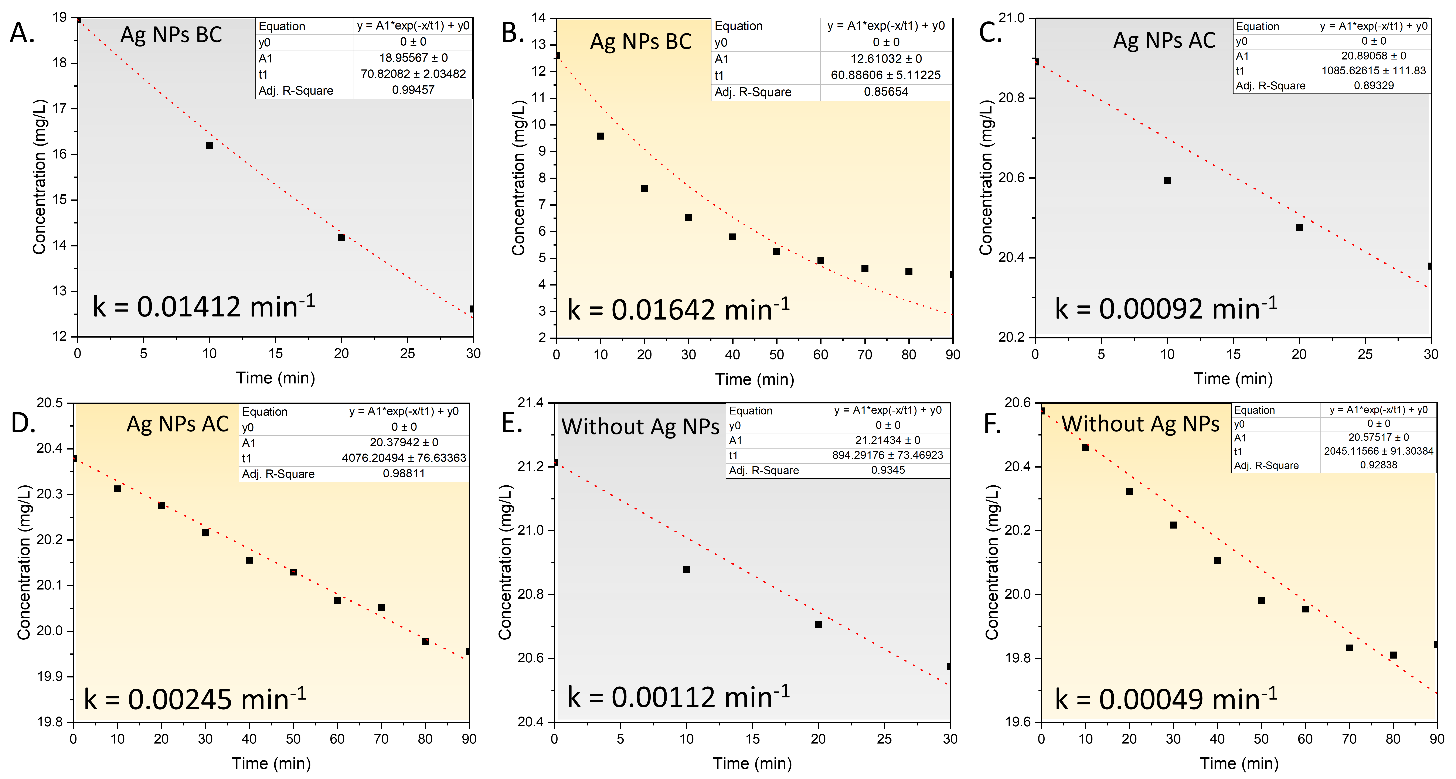


Figure S7 Rate constant (A) Ag NPs BC in the dark, (B) Ag NPs BC in the light, (C) Ag NPs AC in the dark, (D) Ag NPs AC in the light, (E) without Ag NPs in the dark, (F) without Ag NPs in the light. Fitted parameters were calculated based on the dotted red plot.
